# Influence of family cohesion on Chinese adolescents’ engagement in school bullying: A moderated mediation model

**DOI:** 10.3389/fpsyg.2022.1040559

**Published:** 2022-12-08

**Authors:** Xin Chen, Jiarui Jiang, Zuoshan Li, Yue Gong, Jiangli Du

**Affiliations:** ^1^Key Laboratory of Applied Psychology, Chongqing Normal University, Chongqing, China; ^2^School of Teacher Education, Chongqing Normal University, Chongqing, China

**Keywords:** Chinese adolescents, family cohesion, school bullying, self-control, parental monitoring

## Abstract

In this study, a total number of 1,026 Chinese adolescents were surveyed using the cohesion sub-scale of the Family Environment Scale, the Self-control Scale, the Parental Monitoring Questionnaire, and the revised Olweus Bully/Victim Questionnaire to explore the effects of family cohesion on adolescents’ engagement in school bullying and the mechanisms of self-control and parental monitoring in the relationship between them. The results showed that: (1) family cohesion, self-control, and parental monitoring were significantly and negatively related to school bullying; (2) family cohesion directly influenced school bullying and also indirectly influenced school bullying through a mediating effect – self-control; (3) parental monitoring played a moderating role in the path of self-control affecting school bullying. Therefore, to reduce the occurrence of school bullying, it is necessary to strengthen the self-control ability of adolescents and improve the family cohesion environment and maintain a moderate level of parental monitoring. The results of this study revealed the effect of family cohesion on adolescents’ engagement in school bullying and its mechanism of action, which can provide a theoretical basis for preventing and reducing the occurrence of school bullying incidents.

## Introduction

School bullying is a common phenomenon of continued global concern. According to the Global School Violence and Bullying Report released by UNESCO (2017), approximately 246 million children and adolescents worldwide are exposed to various types of school violence and bullying each year, and all children and adolescents are at risk of being involved in school bullying. School bullying refers to repeated instances of physical and psychological persecution and verbal aggression by one or more students against their peers over some time (Olweus, 1994), and it is characterized by disparities in power among peers (Renshaw et al., 2016). Bullying can be divided into physical, verbal, relational, and cyber bullying (Stubbs-Richardson and May, 2020). Each type of bullying can cause multiple internalized and externalized physical and mental health problems for both the bully and the victim. A potential profile-based study explored the patterns of school bullying victimization among contemporary Chinese adolescents, finding that verbal bullying was the most common form of bullying (Xie et al., 2019). Even mild bullying victimization can have long-term adverse effects on the physical and mental health of the victim (Ng et al., 2022). Studies have found that victims of bullying are prone to psychological problems such as anxiety, depression, low self-esteem, suicidal ideation, and physical symptoms such as headaches and insomnia (Campbell et al., 2013). Moreover, adolescent victims of school bullying have shown a decreased positive psychological orientation and subjective well-being, with a higher incidence of emotional and behavioral problems than their peers who are not involved in school bullying (Arslan et al., 2021). For bullies, committing bullying behavior puts them at risk for antisocial personality disorder in the future (Klomek et al., 2009). Olweus (2011) found a longitudinal and prospective association between bullying and later criminal behavior. Bullies have also been found to suffer more psychiatric problems later in life, including depression and panic disorder in adulthood (Copeland et al., 2013; Liu et al., 2021). School bullying poses a huge challenge for school safety and crisis management. Therefore, it is important to explore the influencing factors and mechanisms of school bullying to ensure its prevention and management.

Ecosystem theory assumes that the system and the individual are mutually reinforcing, acting together and influencing the development of the individual, and the.

microsystem is an important part of social-ecological system (Müller, 1992). The microsystem plays an important role in an individual’s development. Composed of family members, peer groups, schools, and neighborhoods, the microsystem is the environment which individuals most directly encounter (Tudge et al., 2009). However, various factors in the family environment can promote or hinder individual growth and adaptation. Family cohesion refers to the degree of emotional closeness between family members (Liu et al., 2014). Students who live in highly close families have fewer internal mental health problems and fewer externalized behaviors (Fosco and Lydon-Staley, 2019). Studies have shown that intimate family interactions have a protective power in relation to bullying, while negative family interactions increase the risk of students becoming involved in such situations (Oliveira et al., 2020). Family cohesion is positively correlated with gregariousness, emotional stability, liveliness, perseverance, and social boldness in personality factors, and negatively correlated with vigilance, apprehension, self-reliance, and tension (Li and Liu, 2012). Families with a high level of intimacy are conducive to the formation of positive personality traits in children. Cohesion in the family environment also promotes the healthy development of individuals’ interaction skills (Liu et al., 2020). The higher the level of family cohesion, the more developed is an individual’s pro-social behavior (Li L. et al., 2020). Since families with a high level of intimacy create a positive atmosphere, adolescents actively seek guidance from their families regarding the adoption of normative behaviors when making moral decisions (Roosa et al., 2011). In contrast, adolescents with a low level of family cohesion lack emotional communication with their parents, so experience interpersonal difficulties. When faced with conflict, they are more likely to respond negatively (Zhao et al., 2011) or even bully others at school. Therefore, it is hypothesized that family cohesion affects the school bullying of adolescents.

Similarly, based on the ecosystem theory of the interaction between individuals and the environment, microsystems and their internal elements also affect the development of individuals’ self-control ability (Putrawan, 2019). The formation of early self-control ability is closely related to family factors, such as family parenting style, family environment, and inter-generational relationships (Malatras and Israel, 2013; Meldrum et al., 2016). A highly cohesive family environment helps to shape an individual’s positive personality (Guo et al., 2021) and positively influences the development and adaptation of self-control (Cho et al., 2018). Thus, family plays an important role in the development of self-control. Self-control theory suggests that individuals with a higher level of self-control have greater autonomy and can regulate their impulses and meet external behavioral standards (King et al., 2011), And self-control, as a regulatory mechanism between an individual’s internal natural impulses and external objective situations (Hofmann et al., 2009), is directly related to problem behaviors such as aggression and bullying (Kim et al., 2022; Sun et al., 2022). In general, people with low self-control are more likely to engage in aggressive behavior than those with high self-control (Liu et al., 2017). In a study on a sample of Nigerian adolescents, regression analysis results confirmed that low self-control might predict adolescents’ experiences of bullying (Fenny, 2021). The study found that individuals with high self-control can inhibit their impulses in a timely manner; actively regulate their cognition, emotion, and behavior according to situational needs and their own intentions; and inhibit adverse and activate positive reactions, so as to avoid harmful behaviors such as aggression and bullying (Zhao et al., 2018). Thus, self-control can be an important predictor of participation in school bullying. Therefore, it is hypothesized that family cohesion influences adolescents’ engagement in school bullying through self-control.

To further explore and gain a deeper understanding of the factors that influence school bullying, we need to examine its moderating mechanisms. According to the theory of social connection, parents’ monitoring of teenagers is an important part of social connection (Hirsch, 1969). and parental monitoring is an important source of social control (Baz Cores and Fernández-Molina, 2022). Teenagers who are monitored by parents will have less opportunities to contact their peers with illegal or problematic behaviors, thus reducing the possibility of these bad behaviors (Tremblay Pouliot and Poulin, 2021). Parents regulate adolescents’ behavior through various means of monitoring, such as attention, guidance, and discipline of activities in which their children are involved (Hou et al., 2017). Effective parental monitoring can reduce problematic behaviors in adolescents, while low-level parental monitoring is significantly associated with problematic behaviors such as aggression, alcohol abuse, and drug abuse (Capaldi et al., 2009; Criss et al., 2015; Moon et al., 2020). The effects of parental monitoring and risk factors on adolescents’ problematic behaviors are not entirely independent, but are combined in a complicated manner. Parental monitoring can act as a positive buffer between risk factors and adolescent problematic behaviors (Ding et al., 2019). Studies have found that poor peer interactions have less impact on the problematic behaviors of adolescents with a high level of parental monitoring than those with a low level of parental monitoring (Hou et al., 2017). Reasonable and moderate parental monitoring can also develop adolescents’ self-control ability and prevent their engagement in criminal acts (Jin et al., 2019). For adolescents with a high level of parental monitoring, their parents have more control over their behaviors and whereabouts, thus promoting standardized behaviors, and adolescents with lower levels of parental monitoring, on the other hand, have more autonomy in their behavior and are more likely to lead to destructive behaviors. Therefore, it is hypothesized that parental monitoring plays a vital role in moderating the path of self-control affecting school bullying.

**Figure 1:**
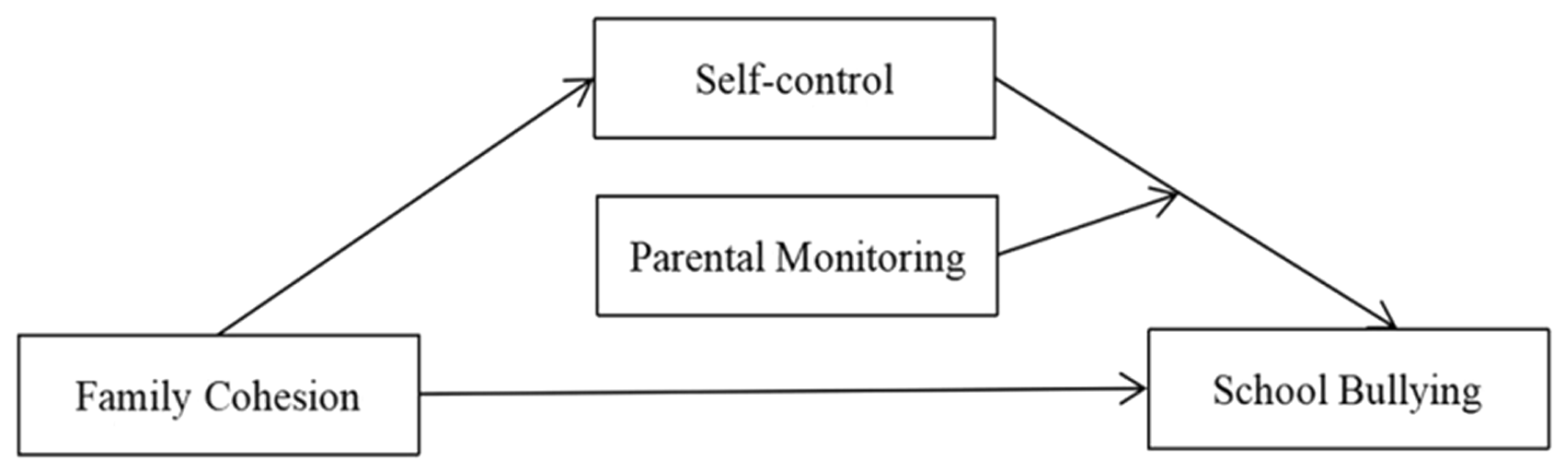
The moderated mediating model.

The development of individual psychological processes like self-control are closely related to the quality of adolescents’s immediate environment, such as the parent–child relationship and family cohesion. In turn, the initial and ongoing contributions of parent and family factors on the development of adolescents’s self-control, influence how they regulate their emotions in the school setting, and hence their potential involvement in bullying. Therefore, the construction of a hypothetical model is shown in Figure 1.

In addition to the above factors, age and gender can also affect adolescents’ engagement in school bullying. Studies have pointed out that school bullying is not based on accidental or certain situations, but on discrimination against peer identity; gender is one of the important elements of an individual’s social identity (Shi, 2017). Boys are significantly more likely to engage in physical violence than girls, so bullying is influenced by gender attributes (Xiao et al., 2020). Young adolescents with incomplete psychological development face a stronger degree of conflict with regard to self-unity and role confusion (Huang, 2007), and are more prone to school bullying. Previous studies have also shown that school bullying of junior high school students is detected at a significantly higher rate than that of senior high school students (Liu et al., 2008), indicating that age is also associated with school bullying. In the relationship between gender and family cohesion, according to Chinese family culture, female socialization is more reflected in her family role and emotional connection. Females keep close ties with their families, and the family cohesion is usually higher than that of males (Liu et al., 2014). Regarding age, previous studies have found that older children reduce their dependence on the family in order to pursue autonomy and adapt to the external environment, so children’s subjective experience of family cohesion decreases during adolescence (Zhai et al., 2021). In terms of self-control, whether there is a gender difference in the development of self-control needs to be further explored. Some studies have shown that there is no gender difference in impulse suppression among young children (Zhang et al., 2020), while Zhang et al. (2012) found that girls have higher self-control ability than boys in adolescents aged 11 to 10. So, the reason for the inconsistent results may be that the sex difference itself is less stable in the early stages of self-control development. Early self-control increases with age. Vazsonyi and Huang (2010) also found that individual self-control increased with age between 4.5 and 10.5 years old. In the period of rapid physiological development in youth, boys are prone to behave impulsively because of hormone levels. Therefore, in terms of parental monitoring, parents have more supervision over the behavior of boys than girls (Deng et al., 2018). With the increase of age, adolescents’ sense of independence will become stronger and they will strive for more autonomous control (Dane et al., 2018). Therefore, parents’ behavior monitoring of adolescents in lower grades is significantly higher than that of adolescents in higher grades. Since age and gender may have significant effects on the results, we attempted to control for the effects of both variables.

In summary, adolescence is a high-frequency period for engagement in school bullying, and it is necessary for us to conduct in-depth research on it. with the support of theoretical research, this study puts forward the following hypotheses: (1) Family cohesion can significantly affect adolescents’ engagement in school bullying; (2) Family cohesion can indirectly influence school bullying through self-control, which plays a mediating effect; (3) Parental monitoring can play a moderating role in the path of self-control affecting school bullying.

## Materials and methods

### Participants and data collection

Stratified random sampling was adopted to cover the cities, suburbs and rural areas of Chongqing and Sichuan. According to the distance from the central city, Chongqing Medical Technology Secondary Vocational School in Nan’an District of Chongqing was selected for urban areas, Chongqing Tongnan Middle School and Sichuan Tongjiang No.3 Middle School were selected for suburban areas, and Chongqing Zhongxian Sanhui Middle School was selected for rural areas. Simple random sampling was conducted in schools in April 2021 to construct research samples and collect questionnaire data from 23 classes. Among them, 6 classes are from urban areas, 10 classes are from suburban areas, and 7 classes are from rural areas. Each class has a maximum of 54 students and a minimum of 31 students. Due to the large number of schools in the district and the large internal differences, stratified random sampling was adopted to reduce the sampling error and improve the representativeness of the sample. In each school, the headteacher assisted in gathering the students for the data collection meeting in the classes, which took the form of an examination, and introduced the researcher and two assistants to students. When the formal questionnaire survey was administered, the headteacher withdrew from the class. The researcher served as the main examiner with two assistants. The main examiner read out the instructions for the test, and the two assistants circulated the classroom to offer guidance about anything unclear in the questionnaire and to prevent discussion among the students. The questionnaires were filled out anonymously. During the survey process, if any respondent rejected answering the questions, the researcher agreed that the respondent would be excluded from the questionnaire survey. This study involving human participants was reviewed and approved by the Local Research Ethics Committee of Chongqing Normal University. Written informed consent to participate in this study was provided by the participants’ legal guardian/next-of-kin.

A total of 1,026 out of 1,100 respondents from the four schools filled in the questionnaire, giving a valid response rate of 93.30%. Among the adolescents, 251 were junior high school students (grades 7–9), 444 were senior high school students (grades 10–12), and 331 were secondary vocational students (grades 10–12); 331 were from cities, 476 were from suburbs and 219 were from rural areas; 424 (41.30%) were males and 602 (58.70%) were females; the age of the respondents ranged from 11 to 20 years old [mean (M) ± standard deviation (SD) = 15.46 ± 1.96].

### Questionnaires

#### Cohesion sub-scale of the family environment scale-Chinese version

We used the cohesion sub-scale (Wang et al., 1999) of the Family Environment Scale-Chinese Version (FES-CV) revised by Fei et al. (1991), The scale consists of nine statements (including “Our family members always give each other the most help and support” and “We feel bored at home”). Respondents were scored according to whether or not they agreed with the statements, with 1 point for “Yes” and 2 points for “No.” The total score was calculated according to a specified computational formula. The higher the total score, the higher the level of family cohesion. The consistency coefficient in this study was 0.75.

### Self-control scale

We used the revised Self-control Scale by Tan and Guo (2008) consisting of five dimensions: resisting temptation, healthy habits, abstaining from entertainment, impulse control, and focusing on work. The scale consists of 19 statements (including “I can resist temptation well” and “It is difficult for me to change bad habits”). A 5-point Likert scale (1 = strongly disagree, 5 = strongly agree) was used to assess the individual students’ self-control ability. The total score of the scale was calculated by adding up the scores of all questions (Questions 1, 5, 11, and 14 were positively scored, and the rest were negatively scored). The higher the total score, the worse the level of self-control. The consistency coefficient in this study was 0.81.

### Parental monitoring questionnaire

Referring to (Lin’s 2001) study, we examined the extent to which parents of adolescents know about their daily lives. The scale consists of eight statements (including “My parents know what I do in my spare time” and “My parents know who my friends are in their spare time”). A 5-point Likert scale (1 = strongly disagree, 5 = strongly agree) was used to assess the extent to which parents monitor their children. The scores of all questions were added up to calculate the total score. The higher the total score, the stricter the parental monitoring of adolescents. The consistency coefficient in this study was 0.85.

### Chinese version of bully/victim questionnaire for middle students

We used the Olweus Bully/Victim Questionnaire established by Olweus (1993) and modified by Zhang and Wu (1999), and selected the Bully sub-scale to obtain data on the frequency of bullying by adolescents toward their classmates. The scale consists of six questions (including “Have you hit, kicked, pushed, bumped or threatened another student this semester?” and “Did you force certain students to give you money, or take or damage something from them this semester?”), In the questionnaire, it was found that a topic of threatening bullying was difficult to be divided into three international methods of implementation, namely, physical bullying, verbal bullying and relational bullying (UNESCO, 2017), so it became a unique category (Chen, 2010). Therefore, the manifestations of bullying in this study are divided into four categories: physical bullying, verbal bullying, relationship bullying, and threats. Threat refers to a bullying method that coerces others through different means to achieve the purpose of bullying. A 5-point Likert scale (1 = “not at all,” 5 = “several times a week”) was used. The total score was calculated by adding up the scores of all questions. The higher the total score, the higher the frequency of bullying by others. The consistency coefficient in this study was 0.91.

### Data analysis

IBM SPSS Statistics 21.0 software was used for data analysis. The following steps were applied: First, we conducted a common method biases test; second, the gender differences of variables were tested and the correlation between variables was analyzed; finally, Model 14 in the PROCESS program compiled by Hayes (2013) was adopted to test the moderated mediation effect. Five thousand samples were taken and the confidence interval was set at 95%. If the confidence interval does not include 0, the effect is significant. Hypothesis testing methods such as T test, analysis of variance and regression were mainly used in this study.

### Control and inspection of common method biases

In this study, data were obtained from questionnaires meaning that common method biases might affect the results. To reduce the impact of error on the research results, Harman’s single-factor test was adopted to examine the results of unrotated factor analysis, the number of factors with characteristic roots greater than one and the cumulative percentage of the first common factor (Podsakoff et al., 2003). The results showed that there were eight factors with characteristic roots greater than 1, and that the variance explanation rate of the first common factor was 15.45%, less than the critical value. Therefore, the current study was not significantly affected by common method biases.

## Results

### Descriptive statistics and correlation analysis

We compared the results of males and females from the four questionnaires on family cohesion, total self-control score, parental monitoring, and school bullying, and adopted the independent samples T-test to determine gender differences in the four variables. The results showed that there were no significant gender differences in family cohesion, total self-control scores, and parental monitoring, while there were significant gender differences in engagement in school bullying, males (M = 7.71) was more likely than females to bully others. These results are shown in Table 1.

**Table 1:** Analysis of differences in variables on gender (*N* = 1,026).

Variable	Male		Female		*t*	*p*	Cohen’s *d*
	*M*	SD	*M*	SD			
Family cohesion	6.58	2.14	6.42	2.41	1.14	0.265	0.07
Total self-control score	50.41	10.78	50.37	10.30	0.07	0.944	0.004
Parental monitoring	25.15	7.25	25.47	6.97	−0.72	0.472	0.05
School bullying	7.71	3.90	6.87	2.43	3.95^***^	0.000	0.31

^*^p < 0.05, ^**^p < 0.01, ^***^p < 0.001.

To facilitate the evaluation of the practical significance of each variable in the gender difference, effect sizes were calculated, which ranged from 0.004 (total self-control score) to 0.31 (school bullying). Cohen (1988) suggested that Cohen’s *d* = 0.2, 0.5, and 0.8 corresponds to small, medium, and large effect sizes, respectively; in the current study, there was a significant gender difference in school bullying, but the effect size was lower than the medium level. This may be because the effect size is affected by sampling, measurement and other objective factors (Ferguson, 2009).

The Pearson correlation test was used to analyze age, family cohesion, parental monitoring, total self-control score, and adolescents’ engagement in school bullying (see Table 2). The data analysis showed that age was significantly negatively related to family cohesion (the correlation coefficient was −0.1, with moderate correlation; the older the age, the lower the family cohesion), and also was significantly negatively related to parental monitoring (the correlation coefficient was −0.23, with moderate correlation; the older the age, the lower the degree of parental monitoring). However, age was significantly positively related to the total self-control score (the correlation coefficient was 0.07, with weak correlation; the older the age, the lower the self-control ability), and has no correlation with adolescents’ engagement in school bullying. Family cohesion was significantly negatively related to adolescents’ engagement in school bullying (the correlation coefficient was −0.15, with moderate correlation; the higher the family cohesion, the lower the school bullying). Family cohesion was significantly positively related to parental monitoring (the correlation coefficient was 0.33, with moderate correlation; the higher the family cohesion, the higher the degree of parental monitoring). Family cohesion was significantly negatively correlated with the total self-control score (the correlation coefficient was −0.21, with moderate correlation; the higher the family cohesion, the lower the total self-control score). Parental monitoring was also significantly negatively correlated with school bullying (the correlation coefficient was −0.11, with moderate correlation; the stricter the parental monitoring, the lower the school bullying). The total self-control score was positively correlated with school bullying (the correlation coefficient was 0.21, with moderate correlation; the stronger the self-control of adolescents, the less the school bullying). However, parental monitoring was insignificantly correlated with self-control.

**Table 2:** Correlation coefficients for each variable (*N* = 1,026).

Variable	1	2	3	4	5
Age	1				
Family cohesion	−0.10^**^	1			
Total self-control score	0.07^*^	−0.21^**^	1		
Parental monitoring	−0.23^**^	0.33^**^	0.00	1	
School bullying	−0.03	−0.15^**^	0.21^**^	−0.11^**^	1

^*^p < 0.05, ^**^p < 0.01, ^***^p < 0.001.

### Family cohesion on school bullying: Examination of the mediating effect of self-control

We first standardized the variables and tested whether there was a mediating relationship between the variables. Based on the mediation test procedures proposed by Wen et al. (2004), the bootstrap method was used to test the relationship between the variables. After controlling gender and age variables, for the indirect path of family cohesion affecting school bullying through the total self-control score, the 95% confidence interval was [−0.07, −0.01], the upper and lower limits were negative, and the 95% confidence interval did not include 0. The effect size was-0.04, and the mediating effect was significant and accounted for 25.00% (the proportion of the effect). In validating the direct effect of family cohesion on school bullying, the 95% confidence interval was [−0.18, −0.06], and the interval also did not contain 0. The effect size was −0.12, and the direct effect was significant and accounted for 75.00% (the proportion of the effect). Without including the total self-control score in the regression equation, the total effect of family cohesion on school bullying was also significant, with an effect size of −0.16. Therefore, the results suggest that family cohesion predicts school bullying in adolescents. The total self-control score can play a significant mediating effect between the two factors, indicating a significant mediating effect of self-control. The specific results are shown in Table 3.

**Table 3:** Decomposition of the total, direct and mediating effects.

Path	Effect size	Boot SE	Boot LLCI	Boot ULCI	Ratio
Family cohesion → Total self-control score → School bullying	−0.04	0.02	−0.07	−0.01	25.00%
Direct effect	−0.12	0.03	−0.18	−0.06	75.00%
Total effect	−0.16	0.03	−0.22	−0.10	

SE, standard error; LLCI, lower limit confidence interval; ULCI, upper limit confidence interval.

### The impact of family cohesion on school bullying: Examination of the moderated mediating model

To explore whether the above mediating effects differ significantly at different levels of parental monitoring, we tested the moderated mediating effects. As shown in Table 4, the test results indicated that family cohesion significantly and negatively predicted the total self-control score (*β* = −0.20, *t* = −6.62, *p* < 0.001), suggesting that family cohesion positively predicted self-control. However, gender and age did not significantly predict the total score of self-control. The predictive effect of family cohesion on school bullying was also significant (*β* = −0.09, *t* = −2.89, *p* < 0.01). Both gender and age had significant predictive effects on school bullying (*β* = −0.24, *t* = −3.95, *p* < 0.001; *β* = −0.04, *t* = −2.44, *p* < 0.05). Moreover, the total self-control score positively predicted school bullying (*β* = 0.18, *t* = 6.02, *p* < 0.001). The same predictive effect of the cross-term of the total self-control score and parental monitoring on school bullying also holds (*β* = 0.12, *t* = 4.96, *p* < 0.001). Parental monitoring moderates the second half of the path of the mediating effect.

**Table 4:** Test of the moderated mediating model.

Regression Equation (*N* = 1,026)		Fit index			Significance	
Outcome variable	Predictor variable	*R*	*R^2^*	*F*	*β*	*t*
Total self-control score	Family cohesion	0.22	0.05	16.61^***^	−0.20	−6.62^***^
	Gender				−0.03	−0.50
	Age				0.03	1.82
School bullying	Family cohesion	0.33	0.11	20.11^***^	−0.09	−2.89^**^
	Gender				−0.24	−3.95^***^
	Age				−0.04	−2.44^*^
	Total self-control score				0.18	6.02^***^
	Parental monitoring				−0.09	−2.72^**^
	Total self-control score × Parental monitoring				0.12	4.96^***^

^*^p < 0.05, ^**^p < 0.01, ^***^p < 0.001.

**Figure 2:**
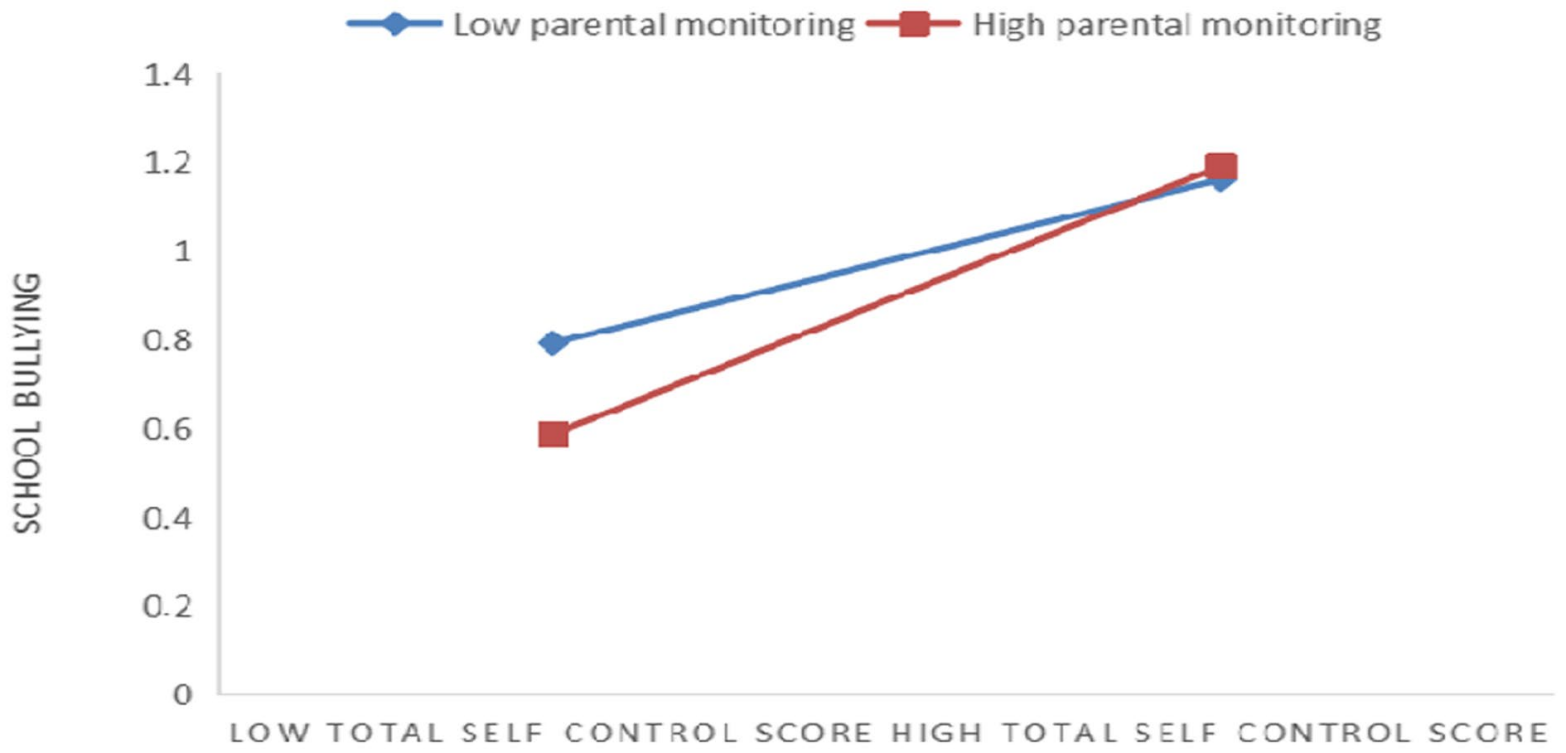
The influence of self-control on school bullying: the moderated effect of parental monitoring. The diamond represents low parental monitoring and the square represents high parental monitoring.

To better explain the effectiveness of moderating effects in the pathway, a simple slope test was conducted to analyze the effect of moderating variables on the pathway (see Figure 2). The data were analyzed by setting the parental monitoring values to low and high groups. The results showed that when parental monitoring moderates the second half of the path of family cohesion–total self-control score–school bullying, the total self-control score is not a significant predictor of school bullying at low parental monitoring levels (M-SD; simple slope = 0.07, *t* = 1.63, *p* > 0.05). Under a high level of parental monitoring (M + SD), the lower the total self-control score and the higher the ability of self-control, the less the school bullying (simple slope = 0.30, *t* = 8.16, *p* < 0.001). This suggests that the higher the intensity of parental monitoring, the more pronounced the inferential effect of self-control on school bullying.

The mediating effect of self-control at different levels of parental monitoring is shown in Table 5. At a low level of parental monitoring (M-SD), the effect size of total self-control score was-0.01, with a 95% bootstrap confidence interval of [−0.05, 0.03], with a positive upper limit confidence interval (ULCI) and negative lower limit confidence interval (LLCI). The interval contained 0, indicating that it was not significant. At a high parental monitoring level (M + SD), the effect size of the total self-control score was-0.06, and the 95% bootstrap confidence interval was [−0.11, −0.03], with a positive ULCI and negative LLCI. The interval did not contain 0, indicating that it was significant. Thus, this suggests that the mediating effect of self-control is more prominent under a high level of parental monitoring.

**Table 5:** The moderated mediating effect.

Intervening variable	Parental monitoring	Effect size	Boot SE	Boot LLCI	Boot ULCI
	M-SD	−0.01	0.02	−0.05	0.03
Total self-control score	M	−0.04	0.01	−0.07	−0.01
	M + SD	−0.06	0.02	−0.11	−0.03

To sum up, in the case of a high level of parental monitoring, adolescents with a higher self-control ability are less likely to engage in school bullying; on the contrary, adolescents with a low self-control ability are more likely to engage in school bullying. A low level of parental monitoring does not moderate the effect of self-control on school bullying. Therefore, increased parental monitoring contributes to a more significant effect of self-control on school bullying.

## Discussion

The current study explored the gender difference test of family cohesion, total self-control score, parental monitoring, and school bullying, as well as the correlation between age and the above four variables. It also explored the direct impact of family cohesion on campus bullying, as well as the intermediary role of self-control and the regulatory role of parental monitoring on the path.

### Test for differences in gender and correlation analysis

The results of this study showed that there were no significant differences in family cohesion, total score of self-control and parental monitoring between genders, while there were significant gender differences in school bullying.

In terms of family cohesion, previous studies have also concluded that there were no significant gender differences in family cohesion (Merkaš and Brajša-Žganec, 2011; Li, 2013). The masculinity of female education and the feminization of male education is a phenomenon that cannot be ignored in family education (Chen and Zhu, 2021), meaning that the differences in behavioral characteristics between males and females gradually fade away. a reasonable assumption is that there is no significant gender difference in family cohesion.

Jo and Bouffard (2014) argue that Gottfredson and Hirschi’s (Gottfredson and Hirschi, 1990) idea of self-control stability can be studied across gender; they believe that males and females should have similar developmental pathways of self-control. Therefore, there is no significant gender difference in self-control. In terms of parental monitoring, previous studies have also confirmed that there is no significant gender difference (Son and Padilla-Walker, 2022). In China, where the number of only children is increasing, parents pay more attention to their children’s family education rather than paying less attention to their children because of their gender. Therefore, we suspect that the absence of significant gender differences in parental monitoring is due to regional and cultural differences as well as the selection of samples.

This study found that males had a higher incidence of bullying than females, which is consistent with existing research findings (Deepshikha and Dalbir, 2018; Li B. L. et al., 2022). A recent study also noted that males were more likely than females to report all forms of bullying (Li X. Q. et al., 2022). Investigate the reason, the first may be related to the physiological factors of males and females, males may show higher aggressive behavior than females because of the influence of adolescent male hormones (Cheng et al., 2020). Second, males’s bullying tend to obtain a dominant position in the group a higher position and target (Patchin and Hinduja, 2010). However, the correlation between adolescent females’s status acquisition goals and bullying behavior is weak (Sijtsema et al., 2009) and due to differences in gender role expectation and socialization process, females than males in relationships tend to show more prosocial target and policy orientation (Almquist et al., 2014). So this leads to a higher incidence of bullying in males than in females.

In the results, there was no significant correlation between age and adolescents’ engagement in school bullying, which was consistent with the previous results (Wang et al., 2022). School bullying exists in a wide age range, throughout the adolescence, and even among adult college students (Gao and Liu, 2018). The results of a survey on bullying in junior high school show that students in grade two are more likely to engage in bullying than students in grade one and grade three (Wang and Liu, 2016). From the perspective of development psychology, junior high school students in grade two are accelerating the development of junior high school self-awareness (Chen et al., 2005). The pursuit of self-control and the constraints of social pressure become the main contradiction faced by students’ psychological adjustment at this time (Longe and Adeyeye, 2019). If the contradiction is not handled properly, it is easy to lead to bullying. It can be seen that it is reasonable that age is not related to participation in campus bullying. In this study, there was no correlation between the total self-control score and parental monitoring for adolescents. An assessment study of eighth-graders found that adolescents with low self-control were more deeply involved in gangs and had an increased likelihood of delinquency, as were adolescents who were not monitored by their parents (Da Na et al., 2010). Self-control is more from the subjective will, is a conscious and hard process (Hoyle, 2006), and is influenced by many aspects of genetics and environment (Mueller et al., 2022), so the correlation between parental monitoring and self-control is not stable, and this result is reasonable.

### Direct effect of family cohesion on school bullying

This study found that family cohesion significantly predicted adolescents’ school bullying, with a negative correlation between family cohesion and school bullying. Adolescents who grew up in intimate families were less likely to engage in school bullying. This suggests that cohesive families can better guide adolescents’ behavioral norms, thus reducing school bullying incidents. Family is the first place for children’s socialization and psychological development (Wang et al., 2020). Some studies have suggested that families that solve problems through indifference or even violence contribute to adolescents having a bullying personality (Su et al., 2016). Adolescents with a low level of family cohesion are more likely to commit delinquent behaviors (Cheng et al., 2016), and seeing high rates of parental conflict and poor parent–child relationships, adolescents are more likely to develop the belief that aggression can solve problems(Xia et al., 2016). Insufficient warmth from family cohesion makes adolescents more prone to aggression when they are feeling less confident, more sensitive, and impulsive (Li X. et al., 2020), which leads to their engagement in school bullying.

### The mediating effect of self-control

The results suggest that family cohesion not only directly influences adolescents’ school bullying, but also indirectly influences it through self-control. In previous studies, some factors in the family were found to influence the cultivation of students’ early self-control ability (Niu et al., 2020; Yun et al., 2020). Moreover, a positive family atmosphere also helps adolescents to develop independent personality traits, to self-regulate and consciously improve their self-control. Therefore, family cohesion can influence individuals’ self-control. Also, previous studies have shown that self-control ability can significantly influence the school bullying of students in rural boarding high schools (Zhang and Zhang, 2019), consistent with the findings of this study. People’s self-control and decision-making are related to a common area of the brain, and the two factors are closely associated (Ren, 2019). Bullying is a decision-making behavior. Adolescents who have high self-control are more likely to restrain themselves from making negative decisions. Therefore, adolescents with an intimate family atmosphere feel a stronger sense of belonging and security in the family, meaning they have a stronger self-control ability. The stronger the self-control, the better they are able to control their behavior and may avoid engagement in school bullying when facing conflict. Since family cohesion affects both school bullying and the formation of self-control ability, self-control affects the emergence of school bullying. Therefore, self-control has a mediating effect between family cohesion and school bullying.

### The moderating effect of parental monitoring

After exploring the mediating effect of self-control in the effect of family cohesion on school bullying, we further examined the moderating effect of parental monitoring on the mediating pathway. The data showed that parental monitoring mainly moderated the second half of the path from family cohesion to self-control and finally to school bullying. This study also clarified that the predictive effect of self-control on school bullying increased with parental monitoring, and that adolescents with a high level of parental monitoring were more likely to be influenced by self-control and engage in school bullying. Continued moderate parental monitoring can indeed enhance children’s social adjustment (Zhang et al., 2011). However, parents who supervise their children severely and over-emphasize behavioral control aggravate the rebelliousness of their children (Jin and Zou, 2013). In the case of a high level of parental monitoring, adolescents with high self-control think calmly in the face of conflict at school and have a lower likelihood of being involved in school bullying. However, adolescents with low self-control may negatively influenced by a high level of parental monitoring. As adolescents with low self-control enter adolescence, sudden changes and discomfort may make them more rebellious (Zhang et al., 2021). The more rebellious adolescents are, the more likely it is that their parents will negatively monitor them (Xu, 2005). At the same time, highly supervisory parents are too restrictive of their children, who have low self-control, which hinders them from communicating with the outside world, thus limiting the development of their behavioral experiences and leading to low social competence, this is more likely to cause negative externalizing problem behavior (Tan et al., 2018). In addition, the more that parents supervise adolescents with low self-control, the more often they have a negative attitude towards adolescents, and they may even use violent education methods, this subconsciously promotes aggressive psychology among adolescents (Chen et al., 2014), who become unfamiliar with the outside world, thus accelerating the development of school bullying. Maintaining moderate parental monitoring is more effective in helping adolescents to reduce or even avoid their problematic behaviors (Deng et al., 2006). Therefore, it is reasonably suggested that the effect of self-control on school bullying is more significant when moderated by a high level of parental monitoring.

## Key findings

The key findings in this study can be summarized as follows:

1. Family cohesion, self-control, and parental monitoring are significantly negatively correlated with school bullying.
2. Family cohesion can directly influence school bullying and indirectly influence school bullying through self-control, with self-control playing a mediating effect.
3. In the model of family cohesion influencing school bullying, self-control played a mediating role, and the second half of the model’s path is moderated by parental monitoring.

## Strengths and limitations

This cross-sectional study was conducted to obtain data on various variables through a questionnaire survey, explore the impact of family cohesion on school bullying behavior through self-control, and verify the moderating effect of parental monitoring. All hypotheses were verified by combining theories with practical data. However, this study also has some limitations. First, cross-sectional studies cannot reveal the causal relationship between family cohesion and school bullying. In future studies, an experimental or longitudinal approach may be used for further analysis. Second, all constructs and outcomes are measured by adolescents’ self-reports, which may exaggerate the relationship between variables. Future studies can combine peer reports, teacher reports and other data collection methods to further enhance the reliability of data. Thirdly, the ecological theoretical model is emphasized in the theoretical discussion of this study. Because there are multiple roles in Microsystems, different roles may have different conceptual understandings of variables, leading to different research results. More related theories should be used in future research to integrate the concept of exploration variables. In addition, the adolescents surveyed in this study come from Chongqing and Sichuan, rather than nationwide. To enhance the reliability of the conclusions, the sample should be expanded for further investigation in future studies.

## Conclusion

School bullying is a safety risk factor and an obstacle for students’ physical and mental health, so it needs to be monitored. The family environment is an important factor that influences the school bullying behavior of adolescents and positively predicts their self-control ability. At high levels of parental monitoring, adolescents with higher self-control are less likely to engage in school bullying behaviors. In contrast, the lower the level of parental monitoring, the more likely it is that adolescents with lower self-control will engage in school bullying behaviors. Therefore, in order to help adolescents reduce school bullying behavior, we can improve their family cohesion, enhance their self-control ability, and maintain moderate parental monitoring.

## Data availability statement

The original contributions presented in the study are included in the article/supplementary material, further inquiries can be directed to the corresponding author.

## Ethics statement

The studies involving human participants were reviewed and approved by The Local Research Ethics Committee of Chongqing Normal University. Written informed consent to participate in this study was provided by the participants' legal guardian/next of kin. Written informed consent was obtained from the individual(s), and minor(s)' legal guardian/next of kin, for the publication of any potentially identifiable images or data included in this article.

## Author contributions

XC was principal author of the manuscript, consulted the literature, and logged the data. ZL was advisor. JJ contributed to data analysis. YG and JD logged the data. All authors contributed to the article and approved the submitted version.

## Funding

This work was supported by Chongqing Education Scientific Planning Project: 2017-GX-119, and was also supported by Comprehensive Education Reform Research Project of Chongqing 2022: 22JGZ03.

## Conflict of interest

The authors declare that the research was conducted in the absence of any commercial or financial relationships that could be construed as a potential conflict of interest.

## Publisher’s note

All claims expressed in this article are solely those of the authors and do not necessarily represent those of their affiliated organizations, or those of the publisher, the editors and the reviewers. Any product that may be evaluated in this article, or claim that may be made by its manufacturer, is not guaranteed or endorsed by the publisher.
